# Supplementary material for: Enhanced Performance of a Visible Light Detector Made with Quasi-Free-Standing Graphene on SiC
Source: Materials (Basel). 2019 Oct 2;12(19):3227. doi: 10.3390/ma12193227 (PMC6804206; doi:10.3390/ma12193227)
Supplement: Supplementary file 1 [file materials-12-03227-s001.pdf]

materials

Article

Enhanced Performance of a Visible Light Detector Made with Quasi-free-standing Graphene on SiC

Xiaomeng Li [1,2], Xiufang Chen [1,2,*], Xiangang Xu [1,2,*], Xiaobo Hu [1,2] and Zhiyuan Zuo [1,3]

[1] State Key Laboratory of Crystal Materials, Shandong University, Jinan 250100, China
[2] Collaborative Innovation Center for Global Energy Interconnection (Shandong), Jinan 250061, China
[3] Advanced Research Center for Optics, Shandong University, Jinan 250100, China

* Correspondence: cxiufang365@126.com (X.C.); xxu@sdu.edu.cn (X.X.)

Received: 16 August 2019; Accepted: 27 September 2019; Published: 10 October 2019

Supplementary Materials

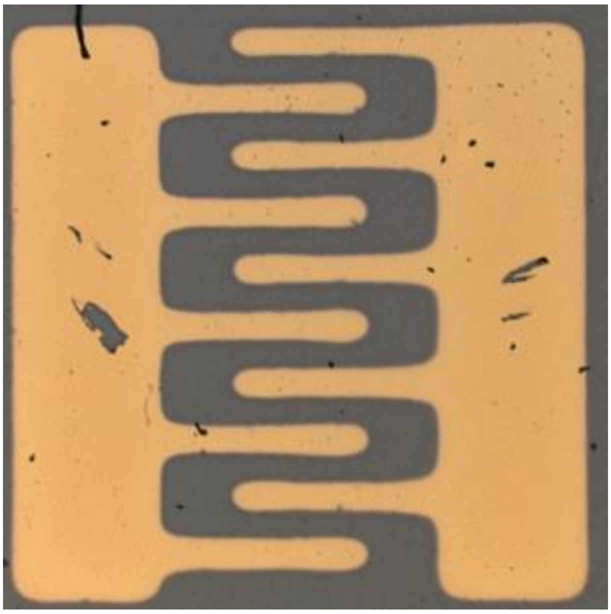

Figure S1. The digital microscopic image of an actual device.

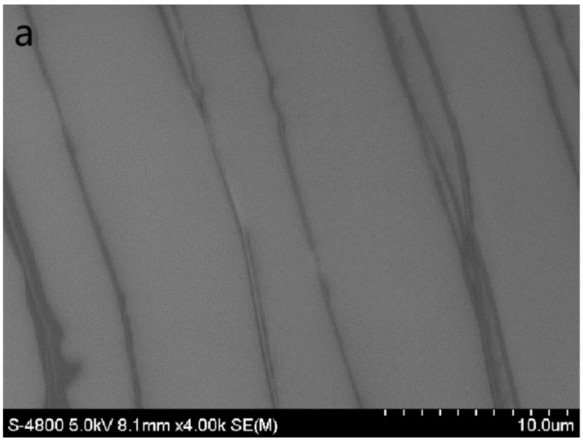

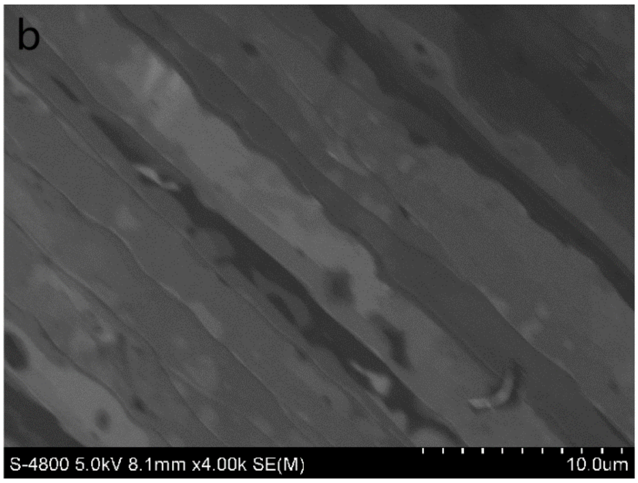

Figure S2. The SEM image of EG sample (**a**) and QFSG (**b**).

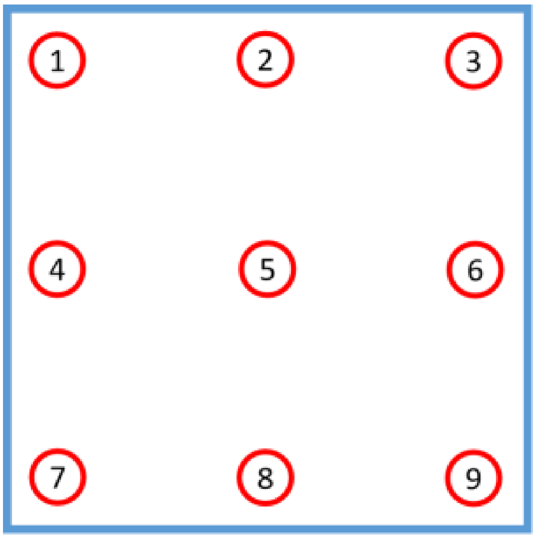

Figure S3. The distribution diagram of the test points for Raman spectra. The circle in the figure is the position of the test point and the number does not represent the test sequence. The horizontal and vertical distance between each two test points is 2 millimeter.

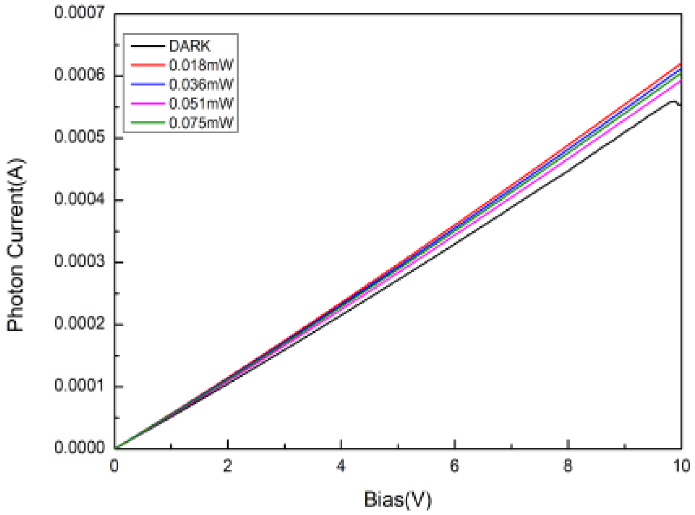

(**a**)

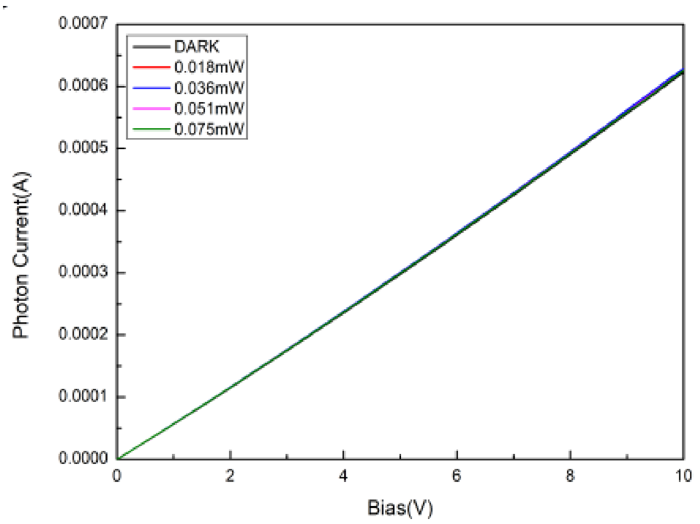

(**b**)

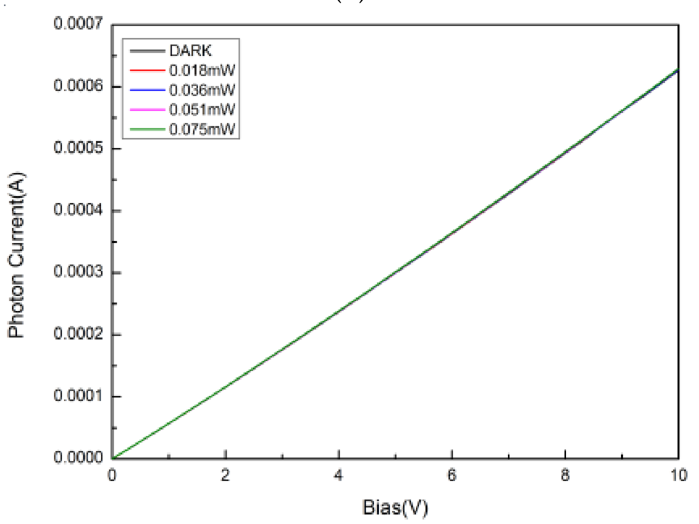

(**c**)

Figure 4. Three photoresponse repeat testing results of the same QFSG sample (**a**–**c**).

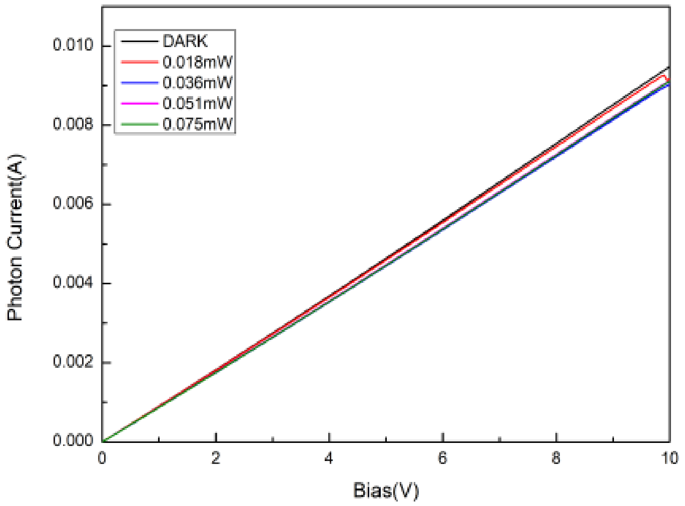

(**a**)

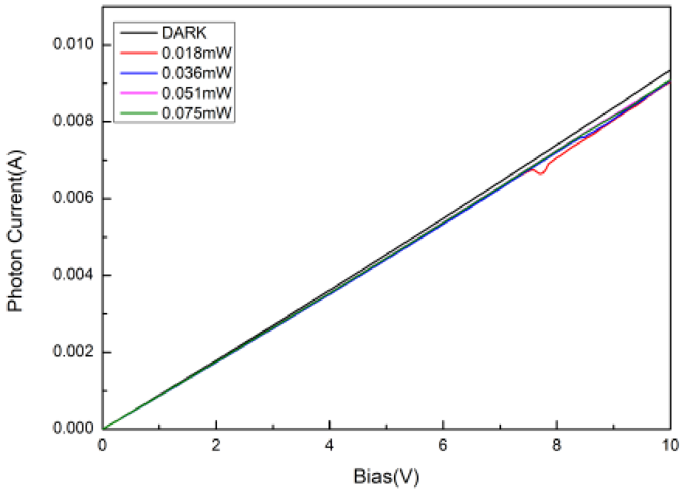

(**b**)

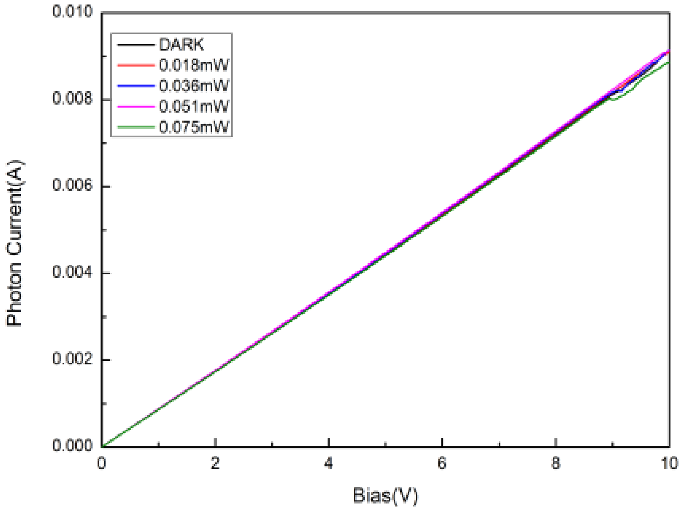

(**c**)

Figure 5. Three ptotoresponse repeat testing results of the same EG sample (**a**–**c**).

© 2019 by the authors. Licensee MDPI, Basel, Switzerland. This article is an open access article distributed under the terms and conditions of the Creative Commons Attribution (CC BY) license (http://creativecommons.org/licenses/by/4.0/).
